# Supplementary material for: SENSES-ASD: a social-emotional nurturing and skill enhancement system for autism spectrum disorder
Source: PeerJ Comput Sci. 2024 Feb 8;10:e1792. doi: 10.7717/peerj-cs.1792 (PMC10909167; doi:10.7717/peerj-cs.1792)
Supplement: Supplemental Information 1 [file peerj-cs-10-1792-s001.zip › templates/emotionWebApp.html]

FACIAL EMOTION RECOGNTION

- IMAGE UPLOAD
- VIDEO UPLOAD
- REALTIME STREAMING

## Upload Image

Click here to select file

## Upload Video

Click here to select file

## Realtime Streaming
